# Supplementary material for: Vaccine Hesitancy among Master’s Degree Students in Nursing and Midwifery: Attitude and Knowledge about Influenza Vaccination
Source: Int J Environ Res Public Health. 2020 Oct 1;17(19):7191. doi: 10.3390/ijerph17197191 (PMC7579204; doi:10.3390/ijerph17197191)
Supplement: Supplementary file 1 [file ijerph-17-07191-s001.pdf]

Survey on influenza vaccination[1]

Università Cattolica del Sacro Cuore di Roma

The purpose of this investigation is to understand your opinion about influenza and flu vaccination in nurses.

We kindly ask you to complete this ANONYMOUS questionnaire.

We ask you to answer all the questions but remember that there are no right or wrong answers.

We thank you in advance for your time.

[1]Bonaccorsi, G.; Santomauro, F.; Porchia, B.; Niccolai, G.; Pellegrino, E.; Bonanni, P. et al. Beliefs and Opinions of Health Care Workers and Students Regarding Influenza and Influenza Vaccination in Tuscany, Central Italy. *Vaccines* **2015**, 3(1), 137-147.

Informed consent agreement for participation in a research study

In accordance with the responsibilities established by the norms of Good Clinical Practice (Legislative Decree 211/2003) of the EU Regulation 2016/679 of the European Parliament and of the Council concerning the protection of individuals with regard to the processing of personal data, as well as the free circulation of such data (hereinafter GDPR EU 2016/679), from General Authorization No. 9/2016 to the processing of personal data carried out for scientific research purposes on December 15, 2016, from General Authorization No. 8/2016 to the processing of genetic data of the December 15, 2016 and by the Guarantor's Resolution for the "Guidelines for the processing of personal data in the clinical trials of medicines" of July 24, 2008 and subsequent amendments, all the information collected with the questionnaires will be used exclusively for scientific research purposes (Article 12, paragraph 1, point d - Legislative Decree 196/2003).

Furthermore, the data collected as part of this investigation are protected by statistical secrecy and therefore cannot be communicated or externalized except in aggregate form, so that no individual reference can be made, and can only be used for statistics purposes (Article 9 of Legislative Decree 6 September 1989, No. 322).

☐ I AGREE **☐ I DO NOT AGREE**

Date ___________________ **Signature _______________________**

SECTION A

Below we list a series of statements, for each you will need to place a cross on the square corresponding to your opinion: "Totally agree", "partially agree", "partially disagree" or "totally disagree".

ITEMS	Totally agree	Partially agree	Partially disagree	Totally disagree
Influenza is a risky disease				
It is better to get sick than to get vaccinated				
The flu vaccine has serious side effects				
The flu vaccine can cause influenza				
The flu vaccine is effective				
The adjuvant increases the effectiveness of the vaccine				
The adjuvant has no serious side effects				
I am against vaccination				
My colleagues do not get vaccinated				
Healthcare professionals must get vaccinated				
I am afraid of needles				
I do not vaccinate so if I get sick, I can stay at home				
It is likely that I transmit the flu				
By vaccinating, I protect myself from the flu				
By vaccinating, I protect my cohabitants / contacts from the flu				
My cohabitants / contacts expect me to be vaccinated against the flu				
The adjuvant has serious side effects				
I know where to get the flu vaccination				
My district promotes flu vaccination				

SECTION B

Below we list a series of statements, for each you will need to place a cross on the square that corresponds to your case.

**1. I got vaccinated against seasonal flu:**	**Yes**	**No**
Last year	☐	☐
Two years ago	☐	☐
Three years ago	☐	☐
**2. I intend to receive seasonal influenza vaccination next year:**	☐	☐

3. If you have not been vaccinated against seasonal flu last year, which was the reason (s)?

	Yes	No
I am not in a risk category		
I was worried about side effects		
The vaccine is not effective		
The vaccine causes the flu		
I never get sick of flu		
The place / time of the vaccination was not suited to my schedule		
I had no time		
I forgot to vaccinate		
I am afraid of needles		
No one informed me about vaccination		
I have never been vaccinated before		

Other, specify ____

4. If you have been vaccinated against seasonal flu last year, which was the reason (s)?

	Yes	No
Not to get the flu		
To protect my cohabitants / contacts		
I get vaccinated every year		
I have been ill with influence in the past		
The place / time for vaccination was appropriate to my commitments		
They advised me to do it		
I felt I had to do it		

Other, specify ____

5. If you have been vaccinated last year, who get you vaccinated?

- The doctor of the hospital vaccination service ☐
- The doctor of the district vaccination service ☐
- The general practitioner ☐
- A colleague ☐
- By myself ☐
- I have not vaccinated last year ☐
- Other: specify ______________________________ ☐

6. If you intend to receive influenza vaccination, who would you like to be vaccinated from?

- The doctor of the hospital vaccination service ☐
- The doctor of the district vaccination service ☐
- The general practitioner ☐
- A colleague ☐
- By myself ☐
- I do not want to get vaccination ☐
- Other: specify ______________________________ ☐

7. The vaccination is to be recommended:

- to people over 65 years ☐
- to pregnant women after the first trimester ☐
- to health professionals ☐
- to oncology patients ☐
- to patients with diabetes and cardiac conditions ☐
- to patients with COPD and renal failure ☐
- to children and healthy young people ☐
- to nobody ☐

8. What kind of vaccine do you prefer?

- Adjuvanted ☐
- Not adjuvanted ☐
- I do not now / is the same ☐
- I do not want to get vaccination ☐

9. What kind of vaccine administration you would like to receive?

- Intramuscular / subcutaneous ☐
- Intradermal ☐
- I do not know / is the same ☐
- I do not want to get vaccination ☐

SECTION C

1. Sociodemographic characteristics

Gender: M ☐ F ☐ Age: _________ years

2. Living conditions:

	Yes	No
Living with children under 9 years old	☐	☐
Living with people over 65 years	☐	☐
Living with people with chronic disease	☐	☐

3. Academic title:

High school ☐

Degree ☐

Post-graduation titles ☐

4. Years of study:

1st ☐

2nd ☐

8. How useful is to implement a campaign aimed at making the hospital's health personnel aware of the flu vaccination? (1 = not at all useful, 10 = totally useful)

1	2	3	4	5	6	7	8	9	10

9. How effective is the hospital's last influenza vaccination campaign? (1 = not at all effective, 10 = totally effective)

1	2	3	4	5	6	7	8	9	10

Thank you for your cooperation!
